# Deceiving HIV: Engineered erythrocytes as cellular decoys

**DOI:** 10.1016/j.omta.2026.201849

**Published:** 2026-09-25

**Authors:** Daniel Stone, Keith R. Jerome

**Affiliations:** 1 Vaccine and Infectious Disease Division, Fred Hutchinson Cancer Center, Seattle, WA, USA; 2 Department of Laboratory Medicine and Pathology, University of Washington, Seattle, WA, USA

## Main text

The human immunodeficiency virus (HIV) remains a major global public health burden. Approximately 40 million people are currently living with HIV, and despite decades of research, complete or functional cures remain elusive. Many approaches have been proposed, but the high mutation rate of HIV and its ability to hide in lymphatic tissues during suppressive drug therapy have thus far proven to be largely insurmountable barriers. In the previous issue of *Molecular Therapy Advances*, Luna and colleagues investigated a novel approach to HIV treatment that uses engineered red blood cells (RBCs) as therapeutic decoys preventing infection and spread.^1^ The authors use a promoterless gene editing approach to drive erythroid-specific expression of a decoy HIV receptor from the *HBA1* (hemoglobin subunit alpha 1) locus in hematopoietic stem and progenitor cells (HSPCs). The highest expression levels were observed using a CD4-glycophorin A (CD4-GPA) fusion, and selective advantage to modified RBC could be conferred by inclusion of a truncated erythropoietin receptor (tEPOR). Using this approach, they produced functional RBCs expressing CD4 decoy receptors that could neutralize HIV *in vitro*, thus preventing infection. This gene editing strategy could potentially be adapted as an *ex vivo* therapy for HIV (Figure 1) and offers an alternative/complimentary approach to other methods currently being developed to prevent, control, or cure HIV infections.^2^

**Figure 1:**
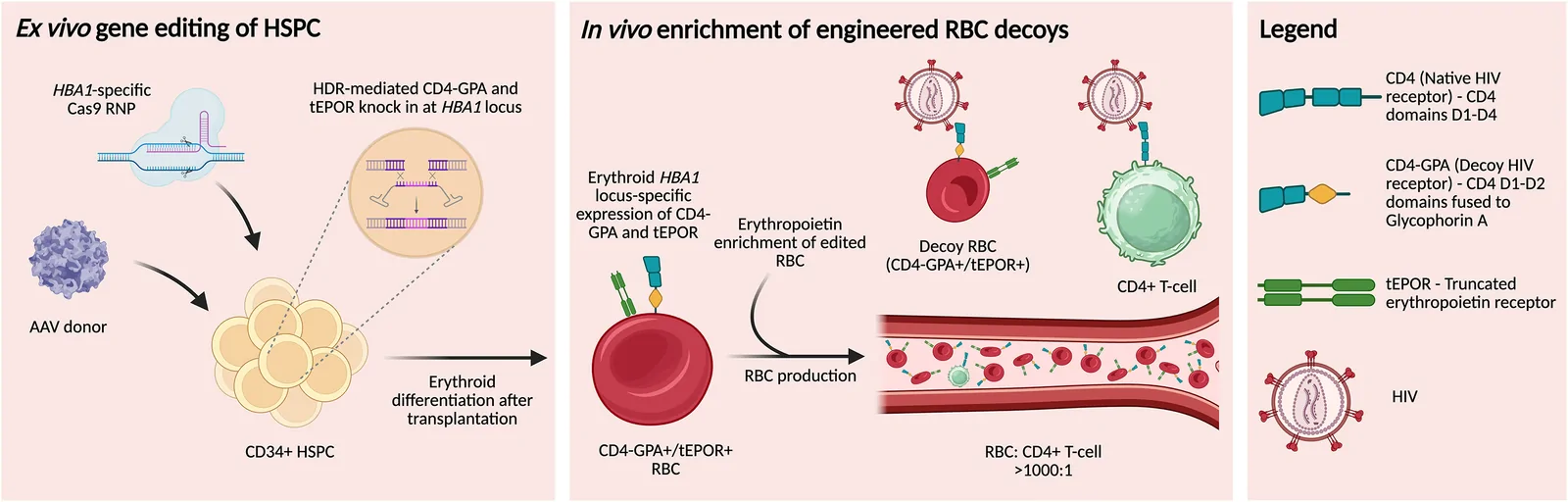
Strategy for the *in vivo* production of HIV-decoy erythrocytes via *ex vivo* gene editing of HSPC AAV, adeno-associated virus; RNP, ribonucleoprotein; HSPCs, hematopoietic stem and progenitor cells; HDR, homology-directed recombination; *HBA1*, hemoglobin subunit alpha 1 gene; RBC, red blood cell; GPA, glycophorin A. Figure created using biorender.com.

The pathway to an HIV cure has proven challenging, with many barriers limiting curative approaches based on vaccination, cell and gene therapy, or transplantation.^3^ To date, only a handful of patients have been cured of HIV, including the initial “Berlin patient” and the most recently reported “Oslo patient”. All received myeloablative allogeneic stem cell transplants as treatment for blood-derived cancers, mostly with donor stem cells containing the CCR5Δ32 mutation in the HIV CCR5 co-receptor.^4^^,^^5^ While this approach to HIV cure appears reproducible, allogeneic stem cell transplantation remains expensive, complicated, and dangerous and is not considered appropriate for patients with HIV controlled by antiviral therapy that do not require treatment for complications like cancer. Thus, safer methods to achieve either a complete or functional cure for HIV are still needed.

In this article, the authors advance a previously described approach to HIV therapy from Hoffmann et al. that introduced cellular decoys that neutralize HIV into HSPC-derived erythroid progenitor cells prior to differentiation into RBCs.^6^ They build upon this work and their experience engineering HSPCs to promote erythropoiesis,^7^ developing a strategy that differs by: (1) introducing CD4 decoy receptor expression in RBCs via a Cas9-RNP/AAV6 gene editing approach that directly modifies CD34^+^ HSPCs prior to erythroid differentiation rather than through lentivirus vector transduction of short-term erythroid progenitor cells derived from *in vitro* differentiation of CD34^+^ HSPCs, (2) using a promoterless construct that targets the *HBA1* gene locus (a non-essential gene due to production of hemoglobin alpha from the *HBA2* gene) via homology-directed recombination (HDR) to enable erythroid-specific CD4 decoy receptor expression in HSPCs, and (3) incorporating a tEPOR in their knock-in constructs to enable enrichment of CD4^+^/tEPOR^+^ expressing cells during erythroid differentiation.^8^ Importantly, this approach cannot provide a complete HIV cure as it does not remove the existing latent HIV reservoir, but it does offer the potential for a functional cure by preventing ongoing spread of HIV.

The Cas9-RNP/AAV6 gene-editing approach to HSPC modification allows for precise integration of CD4 decoy receptors without the potential risks associated with lentivirus transduction, such as insertional mutagenesis or insertion site-related transcriptional silencing. The authors achieved knock-in efficiencies of ∼30% at *HBA1* in both cord blood- and peripheral blood-derived CD34^+^ HSPCs, and CD4 expression levels were higher on engineered RBCs than on naive or activated CD4^+^ T cells. While Hoffman and colleagues previously used lentivirus transduction of erythroid progenitors to attain higher levels of gene modification than the initial levels reported here, the incorporation of tEPOR into the AAV6 donor construct enabled further enrichment to >80% engineered RBCs after 14 days of differentiation.

A successful RBC-based HIV decoy receptor strategy will require gene modification of HSPCs or erythroid progenitor cells without impacting their differentiation potential or the basic physiological functions of circulating RBCs. Therefore, the authors evaluated gene-modified HSPCs and RBCs using a number of assays. They found that gene editing does not impede myelo-erythroid differentiation of HSPCs in a colony forming unit assay and that erythroid differentiation-specific transcriptional programs in CD4-RBCs are not impacted by gene editing via RNA sequencing (RNA-seq). Using flow cytometry, they demonstrated that gene-modified RBCs are phenotypically CD71, CD36, and Band3 positive and α4-integrin negative. CD4-RBCs were also negative for the enucleation marker DRAQ5. In an elongation assay, CD4-RBCs showed no evidence of sickling, rigidity, or impaired deformability when compared to mock edited HSPC-derived RBCs. CD4-RBCs also produced adult and fetal hemoglobin at levels comparable to or greater than mock edited HSPC-derived RBCs. So far, no abnormalities have been seen in the properties of RBCs derived from gene-edited HSPCs *ex vivo*, however, future studies may need to follow long-term clonal behavior and hematologic safety of cells given a selective advantage following tEPOR-mediated expansion *in vivo*.

After first demonstrating that immortalized K562 cells expressing their CD4-GPA decoy receptor can neutralize an HIV pseudovirus containing the TRO11 clade B envelope in a TZM-bl assay at a concentration of 6 × 10^7^ cells/mL, the authors assessed the ability of HSPC-derived CD4-RBCs to inhibit HIV infection using pseudoviruses packaged with either the TRO11 or CNE55 envelopes using the TZM-bl assay. CD4-GPA^+^/tEPOR^+^ RBCs were able to efficiently neutralize both pseudoviruses at similar levels using the highest cell concentration tested (6 × 10^7^ cells/mL). While the authors considered this level of neutralization relatively inefficient, importantly, this concentration is approximately 1.2% of the RBC concentration in blood and suggests that neutralization of HIV might be more complete *in vivo* if higher ratios of engineered to naive RBC are obtained. Since donor-to-donor variations in HSPC gene editing could also be seen in a treatment setting, it also offers potential that efficient HIV neutralization could be achieved *in vivo* with comparably lower levels of gene editing and engineered RBC chimerism than reported here. More testing in a suitable model containing all blood components and lymphoid tissues will be needed to evaluate the efficiency of decoy RBC neutralization.

If further optimized, the promising cellular decoy approach described here could be employed to control ongoing replication and disease progression in HIV^+^ individuals. It would likely be simpler and safer than allogeneic bone marrow transplantation, as it would use autologous HSPCs, thus reducing the risk of graft versus host disease and would likely not require complete myeloablation or donor chimerism to achieve sufficient levels of decoy RBC in blood. Nevertheless, the *ex vivo* manipulation, expansion, and transplantation of engineered HSPCs required is not trivial or inexpensive, and further work is needed to simplify and validate this cellular decoy approach. As a next step toward therapeutic validation, animal studies should be performed to determine whether engineered HSPCs can efficiently and longitudinally engraft and differentiate into decoy receptor expressing RBC *in vivo*, and what levels of engineered RBCs are required for the neutralization of HIV, although this may prove challenging. HIV replication is species-specific, and most humanized mouse models that support HIV replication and/or HSPC transplantation and human hematopoiesis do not support human erythropoiesis due to macrophage-mediated phagocytosis of erythrocytes. Thus, future validation may need to be performed in simian-human immunodeficiency virus-infected primates, or in new mouse strains that support human erythropoiesis following CD34^+^ HSPC transplantation, albeit sub-optimally, with RBC engraftment levels approaching 10%–12% human RBCs in blood.^9^^,^^10^ Ultimately, without further testing in a model of blood-borne HIV infection that contains all the cell types found in human blood at appropriate ratios and concentrations it will be hard to know the true promise of a decoy RBC approach to HIV therapy.

## Declaration of interests

K.R.J. is a founder and holds equity in Caladan Therapeutics, has been a paid advisor and holds equity in Excision Biosciences, and has participated in sponsored research agreements with Excision Biosciences and Emendo Biotherapeutics. D.S. has participated in sponsored research agreements with Excision Biosciences and Emendo Biotherapeutics.
